# Insights into the Use of Eco-Friendly Synergists in Resistance Management of *Leptinotarsa decemlineata* (Coleoptera: Chrysomelidae)

**DOI:** 10.3390/insects13090846

**Published:** 2022-09-16

**Authors:** Rana Muhammad Kaleem Ullah, Ayhan Gökçe, Allah Bakhsh, Muhammad Salim, Hai Yan Wu, Muhammad Nadir Naqqash

**Affiliations:** 1 Guangxi Key Laboratory of Agric-Environment and Agric-Products Safety, National Demonstration Center for Experimental Plant Science Education, Agricultural College of Guangxi University, Nanning 530004, China; 2 Department of Plant Production & Technologies, Faculty of Agricultural Sciences and Technologies, Niğ de Omer Halisdemir University, Niğde 51200, Turkey; 3 Institute of Plant Protection, MNS—University of Agriculture Multan Pakistan, Multan 60000, Pakistan

**Keywords:** *Leptinotarsa decemlineata*, environmental concerns, input cost, synergists, resistance management

## Abstract

**Simple Summary:**

The discovery of insecticides requires time and has a high investment cost. However, injudicious use of insecticides has resulted in insect pest resistance and pest resurgence. These factors limit the market life of insecticides. To cope with this problem, synergists working as blockers of detoxification enzymes can provide a unique solution in resistance management. Owing to problems associated with chemical synergists, plant-based and RNAi-based synergists are much safer and easier to develop against insects. In fact, the Colorado potato beetle (CPB) has a unique detoxification mechanism due to its co-evolution with Solanaceous plants. This review is about how synergists can be used to deal with the resistance management of the CPB and will be helpful for researchers devising unique pest management strategies for other insect pests.

**Abstract:**

The Colorado potato beetle (CPB), *Leptinotarsa decemlineata* (Say), is the most notorious insect pest of potato globally. Injudicious use of insecticides for management of this pest has resulted in resistance to all major groups of insecticides along with many human, animal health, and environmental concerns. Additionally, the input cost of insecticide development/discovery is markedly increasing because each year thousands of chemicals are produced and tested for their insecticidal properties, requiring billions of dollars. For the management of resistance in insect pests, synergists can play a pivotal role by reducing the application dose of most insecticides. These eco-friendly synergists can be classified into two types: plant-based synergists and RNAi-based synergists. The use of plant-based and RNAi-based synergists in resistance management of insect pests can give promising results with lesser environmental side effects. This review summarizes the resistance status of CPB and discusses the potential advantage of plant-based and RNAi-based synergists for CPB resistance management. It will motivate researchers to further investigate the techniques of using plant- and RNAi-based synergists in combination with insecticides.

## 1. Introduction

The Colorado potato beetle (CPB), *Leptinotarsa decemlineata* (Say) (Coleoptera: Chrysomelidae), is the most damaging insect pest of potato and many other members of Solanaceae globally [1,2]. It originated in Mexico and south-west America; and gained the importance as a notorious global insect pest [3]. Average potato leaf consumption by an adult beetle is around 40 cm^2^, while its larval stage eats 9.65 cm^2^ of leaves daily [4]. Defoliation in potato plants due to the CPB results in an annual yield reduction of around 30–50%, or sometimes no economic yield can be obtained [5].

Chemical control is the method mostly preferred by potato growers for the control of the CBP. Currently, chemical control is the most effective and promising way to manage the CPB in potato fields. However, high selection pressure due to the use of chemical control has resulted in CPB resistance against a variety of synthetic insecticides [6,7]. The actual reason behind CPB insecticide resistance development is the evolution of this insect with diverse phytochemicals in Solanaceous plants, which has been combined with heavy insecticide applications every year for its control since 1864 [8]. Since the 1950s, this notorious insect has become resistant to approximately every chemical used for its control. Therefore, alternative strategies for CPB management should be explored [9]. In the pesticide industry, tremendous numbers of chemicals are synthesized and evaluated annually for their insecticidal properties. However, due to the increasing resistance development of the CPB, there has been an increase in input costs and a decrease in the advantage from insecticide applications [10]. To address the issues of insect pests and insecticide resistance, new scientific discoveries are required [11,12].

Synergists have a significant role in hindering metabolic resistance by inhibiting detoxification enzymes. Synergists/chemicals are added to the insecticides to increase their toxicity, when given in sub-lethal doses [13]. Synergists have been utilized in different ways since the first report on enhanced insecticidal activity of pyrethrum after the addition of the natural synergist “sesamin” [14]. Metcalf [15] defined the term synergist as “The component of a mixture that is not toxic alone, at the rate of treatment, but enhances the lethality of the insecticide being applied with it”.

Chemical synergists have been used commercially for more than 60 years and have contributed significantly to the efficacy of insecticides, particularly when problems of resistance have arisen. Common examples of chemical synergists include piperonyl butoxide (PBO), diethyl maleate (DEM), verapamil (VER) for ABC transporters, and S,S,S-tributyl phosphorotrithioate (DEF) [13,14,15]. For the management of resistance in insect pests the selection of eco-friendly synergists is very important. These eco-friendly synergists can be divided into two main categories, i.e., plant-based synergists and RNAi-based synergists. The use of plant-based products and RNAi techniques in insect pest control is gaining importance due to lesser environmental hazards, low mammalian toxicity, specificity, and quicker degradation in the environment. The synergistic approach of the use of botanicals and RNAi separately and in combination against the CPB can be considered a potential eco-friendly insect management strategy. This review article summarizes the current status of resistance in the CPB and how the use of environmentally friendly synergists can improve the efficacy of insecticides.

## 2. Current Status of Resistance in the CPB

The CPB was the first insect on which insecticide spray was used on a larger scale in 1864 [8]. Resistance can be increased 100-fold against insecticides, under selection pressure, after only three generations [16]. It developed resistance to neonicotinoids within 2 years of commercialization on Long Island, USA [17]. Organochlorine resistance has been reported to increase up to 220× [18], while the level of resistance to organophosphates can enhance to 252.1× [19]. Carbamate resistance has been reported to surge up to 18×. Resistance to pyrethroids can enhance to as much as 2749-fold in the field strain [6,20]. Resistance to newer insecticides has also been well reported, for example, imidacloprid resistance can enhance up to 310×, while spinosad can enhance to 7.6× [21]. The levels of resistance to chlorantraniliprole can enhance to 4.89× [22]. Additionally, resistance to BT Cry 3A has also been reported [23].

### Genetic Basis of Resistance

Solanaceae plants have higher levels of plant secondary metabolites (glycoalkaloids) and the co-evolution of the CPB with these plants has naturally enhanced its ability to survive under the worst conditions of selection pressure [24]. Like other resistant insect pests, the CPB also uses various mechanisms of resistance to survive the insecticide treatment. Mechanisms of resistance are very diverse because of their exposure to a variety of plant metabolites and synthetic chemicals. The major mechanisms of insecticide resistance in the CPB include decreased penetration [25]; target-site insensitivity [19]; enhanced excretion [26]; and sequestration and complex of detoxification enzymes including carboxylesterases, glutathione-S-transferases (GSTs), and monooxygenases. Whereas, sequestration of compounds needs selective transport and storage, which limits the toxin affecting the normal physiological processes of the insect [27]. Similarly, most reported cases of “behavioral resistance” to insecticides were aversion behaviors, whether they are learnt or innate. Reduced penetration and enhanced excretion work in conjunction with other resistance mechanisms [25,26]. Among them, metabolic resistance has been well studied and is considered to be a derivative of an inherited capability to detoxify toxins present in food [28].

The organophosphate-resistant CPB usually contains point mutations from serine to glycine in the acetylcholinesterase (*AChE*) gene [29]. Around 45 different kinds of mutations have been reported in four field populations which were contributing to *AChE* insensitivity [19]. Additionally, particular point mutations such as I392T, S291G, and R30K found in carbamate and organophosphate-resistant CPBs were discovered via site-directed mutagenesis [7].

Mutations, i.e., L1014F and S291G in LdVssc1 and acetylcholine esterase, result in resistance to pyrethroids [30]. Partial resistance to carbamates has been reported due to the mutation *AChE* termed as S291G, while point mutations in LdVscc1 termed as L1014F confer pyrethroids resistance [22].

**Figure 1:**
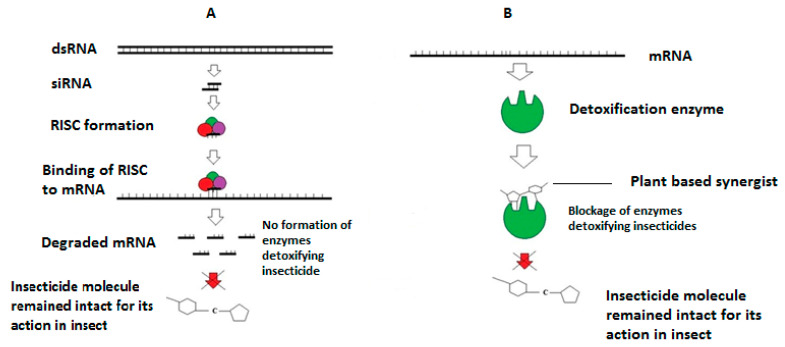
Difference between (**A**) siRNA-mediated synergism due to mRNA degradation and hence no detoxification of insecticides and (**B**) Plant-based synergism due to physical blockage of detoxification enzymes resulting in no detoxification of insecticide(s). Adapted from Cooper et al. (2018) after changes.

The highly diverse mechanisms of resistance in the CPB to approximately all the introduced insecticides for its control are the key reason to explore new ways of control. In this regard, synergists can help researchers in the eco-friendly management of the CPB with reduced use of insecticides. A simple graphical representation regarding the mechanism of resistance and how synergists will help in overcoming that resistance is shown in Figure 1.

## 3. Plant-Based Synergists

The majority of synergists, which are similar in composition to insecticides, physically block the metabolic systems so the molecules of the insecticide molecules cannot be detoxified [31,32].

Many plant extracts can potentially synergize with conventional insecticides. Plant-based synergists received the attention of researchers in 1974 [33]. These plant extracts belong to essential oils, alkaloids, phenolics, terpenes, and many other secondary metabolites [33,34].

### 3.1. Plant Secondary Metabolites

#### 3.1.1. Plant Oils

**Figure 2:**
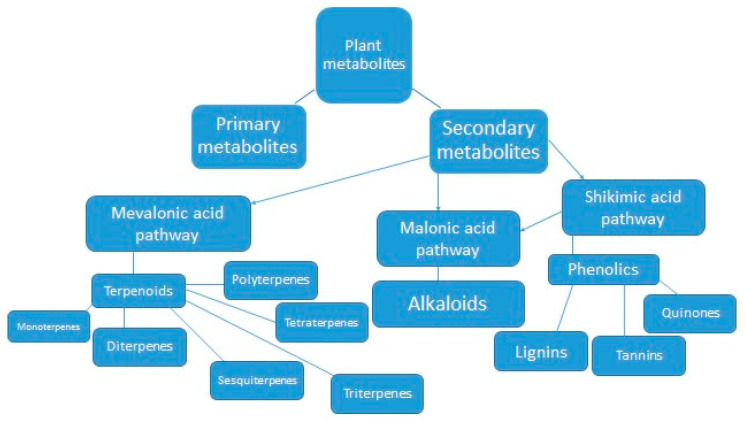
Formation of different kinds of secondary metabolites in plants, adapted from Mendoza and Silva [36] after modifications.

Plant metabolites are broadly divided into two categories, i.e., primary metabolites (playing roles in basic plant functions) and secondary metabolites (produced as by-products of subsidiary pathways and playing roles in plant defense mechanisms). Aromatic plants biosynthesize essential oils as secondary metabolites. Most of them are volatile, chained biochemicals with a peculiar odor. Oils are being used as analgesic, sedative, antimicrobial, anti-inflammatory, and locally anesthetic remedies [35] (Figure 2).

There are a number of vegetable oils that can be used as synergists to increase the efficacy of a number of insecticides. The most common toxic and non-toxic oils that can be used as synergists with conventional insecticides include sesame oil, karanja oil, neem oil, citronella, etc. [37,38].

Seed extracts of dill plants (*Anethum graveolens* L.) have been reported to possess a synergistic effect for organophosphates and carbamates against some insect pests. Aerial parts of this plant usually contain d-carvone; both shoots and roots possess myristicin; while dillapiol and apiol are found only in roots [39]. Plant extracts from aerial parts of the dill plant have been reported to contain more active synergists as compared to root extracts. This compound is capable of increasing the toxicity of carbamates to insect pests. However, the root extracts rich in apiol, dillapiole, and myristicin have been found to be more effective synergists of the synthetic insecticides than d-carvone even at very small doses [33]. These findings may help us understand which plant species may need smaller doses of insecticides in insect pest management because of the presence of natural synergists in their tissues. It could also help pest managers to find ways to use a mix of these extracts and conventional insecticides to get rid of pests more effectively [40].

Pongam oil (extracted from *Pongamia glabra* L.) and its constituents exhibit synergistic properties. The toxicity of synthetic insecticides such as Isolan, Pyrolan, carbaryl, endrin, or heptachlor can be significantly enhanced by adding the oil of *P. glabra* and its constituents, i.e., karanjin and pongamol, against adult houseflies (*Musca domestica* L.) and the cotton stainer, *Dysdercus cingulatus* F. [37]. Vegetable oils of *Sesamum indicum* L., *Millettia pinnata* L., *Azadirachta indica* L., and *Pelargonium citrosum* L. can successfully increase the toxicity of pyrethroids to a higher extent as the synthetic synergists including PBO and DEF increase the toxicity of pyrethroids against beetles [41]. Some essential oils such as pongam oil, neem oil, and citronella oil can significantly enhance the toxicity of pyrethroids against lepidopterans just like commercial synergists [38]. Some essential oils such as Dillapiole (an essential oil commonly extracted from dill plant and fennel plant roots and also a member of benzodioxoles) can be used to increase the toxicity of several botanical insecticides. Dillapiole significantly synergized the activity of neem, rotenone, and toosendanin against lepidopteran larvae. However, a negative interaction with growth-inhibiting effects was observed [42].

Various vegetable oils obtained from *Linum usitatissimum* L., *Gossypium hirsutum* L., *Hibiscus sabdariffa* L., *Carthamus tinctorius* L., *Sesamum indicum* L., and *Pongamia pinnata* Pierre have been reported to synergize pyrethroids (fenvalerate, deltamethrin, and cypermethrin) against notorious insect pests such as the diamondback moth (*Plutella xylostella* L.) [43]. Nanoemulsions of essential oils have been reported to enhance the synergism of some botanicals. Nanoemulsions of eucalyptus oil containing jatropha and karanja aqueous filtrate can induce 88–100% mortality against beetles within 24 h [44] as shown in Table 1.

#### 3.1.2. Alkaloids

Alkaloids, derivatives of amino acids, are a chemically diverse group of nitrogen-containing compounds with low molecular weight. These secondary metabolites are biosynthesized by 20% of plant species. They have a significant role in plant defense against insect pests and pathogens [45] (Figure 2).

Three taxoids (terpenoids isolated from *Taxus* spp.) were distilled from an extract of foliage from English yew (*Taxus baccata* L.) which synergized the pyrethroids against the black vine weevil (*Otiorhynchus sulcatus*) [46].

Root extracts of *Vepris uguenensis* Engl. contain a known compound alkaloid (flindersiamine) whose fractions can significantly increase the toxicity of pyrethrins against dipterans [47].

Some newly discovered phytochemicals have remarkable potential for synergizing insecticides due to their ability to inhibit GST and esterase inhibitors [48]. Taxifolin (a flavonoid) significantly enhanced the toxicity of azinphos-methyl against insecticide-resistant CPB. Greater strength of inhibiting estrases was observed in the case of taxifolin as compared to the commercial esterase inhibitor “DEF”. Research has demonstrated that flavonoid compounds can inhibit estrases activity both in vitro and in vivo, which can be the main cause of insecticide synergism when applied to resistant CPB strains [49] (Table 1).

#### 3.1.3. Phenolics

Phenolic compounds are necessary for plant pigmentation, growth, reproduction, and a variety of other functions [50,51]. They are potential chemical barriers against foreign invaders such as bacteria, fungi, nematodes, plant-feeding insects, and other herbivores [52,53], as shown in Figure 2.

The glutathione level was affected by the combined treatment of phenolics with a saponin and an alkaloid, supporting the fact that a ‘‘combination’’ of secondary metabolites can be more useful for plant protection. Test larvae which were exposed to ellagitannins and phenolics showed a greatly enhanced reactive oxygen level in their midgut [54] (Table 1).

#### 3.1.4. Terpenes

Terpenoids contain the highest number of plant secondary metabolites, reaching around 40,000 members, making an impressive example of the evolution of chemical diversity in plants. The simplicity of formation of variously sized molecules has made them an ecologically successful class among secondary metabolites. Classification of terpenoids is usually based on the number of carbon atoms in their skeleton, e.g., monoterpenes, sesquiterpenes, diterpenes, and triterpenes [55] (Figure 2).

Volatile terpenoids are important either as constitutive compounds or biotic stress-induced allelochemicals in the plant defense mechanism. Monoterpene volatiles released by the leaves of *Chrysanthemum morifolium* Ramat have repellent activities and deterrent effects against insect pests [56,57]. Furthermore, glandular trichomes also store volatile terpenoids, which act as insect repellents, e.g., sesquiterpenes found in the trichomes of wild tomato are strong repellents to homopterans [58]. The monoterpene (+)-3-carene are connected with insecticidal activity of *Picea sitchensis* Carr on the Engelmann spruce weevil (*Pissodes strobi*) [59]. Additionally, they also interact with herbivores and their natural enemies, constitutive and prompted mixtures can play role in interspecific, intraspecific, and even via “alarm” signals that may activate the defensive mechanism in adjacent plants [60,61]. Mixtures of terpenes with each other have been proven to synergize each other against insects. Nonvolatile terpenoids also work in assistance with volatile terpenoids for plant defense, e.g., glycosides of geranyllinalool in wild tobacco may act as potent antifeedants [60,61,62].

Complex essential oils have minor proportions of natural synergists. The synergism of *trans*-anethole with thymol, citronellal, and R-terpineol has been reported against lepidopterans. Various biochemicals mixtures have been synthesized and tried as effective control agents, on the basis of results. Some mixtures revealed promising synergistic effects. The result of this research can be further used for the commercial production of terpene-based synergists [63] (Table 1).

#### 3.1.5. Miscellaneous

A number of plant extracts can also be used for their synergistic activity. Some of the common examples are discussed below:

Root extracts of surattense nightshade (*Solanum xanthocarpum* L.) synergized the larvicidal activity of cypermethrin against mosquito larvae [64]. A synergistic effect of ethanol extracts of the leaves of *Melia azedarach* L. and *Jatropha gossypifolia* L. with cypermethrin against lepidopteran larvae has been proven. General esterase and acetylcholinesterase activities were inhibited by both tested extracts [26] (Table 1).

However, only a small number of botanicals are employed in agriculture in the industrialized world, and there are few prospects for the commercial development of new plant-based synergists, despite the growing quantity of scientific literature showing the bioactivity of plant derivatives against arthropod pests [65,66]. However, the production, preparation, or usage logistics of plant-based synergists can mitigate against their utilization despite their bio-activity against pests. As a matter of fact, the majority of these synergists are targeting chewing insect pests or beetles. Thus, they can be successfully deployed against the CPB for its effective management [65].

## 4. Use of RNAi as Synergists

The use of RNAi as eco-friendly synergists along with insecticides will not only increase the efficacy and life of these insecticides but it will be a step towards a lesser polluted environment [67]. Successful knockdown of resistance-conferring genes has been reported various times in the CPB and many other insect pests. Some important targets deployed specifically against the CPB and explored to date are presented below.

Juvenile hormone (JH) has become established as the principal hormone controlling reproduction in female insects. Additionally, signaling and nutrient-sensing pathways have highlighted the role of JH in insects [68]. In the JH biosynthesis pathway, S-adenosyl-L-methionine-mediated methylation of JH produces S-adenosyl-L-homocysteine (AdoHcy) [69,70]. Rapid removal of AdoHcy is necessary for the JH production in insects [71]. Its expression can be found in all growth stages. A feeding bioassay with dsRNA targeting *LdSAHase* significantly downregulated the expression of *LdSAHase* and *LdKr-h1*mRNA, decreased JH titer, and resulted in significant mortality of exposed larvae, and a decrease in the formation of pupae and emergence of adults. Additionally, silencing of *LdSAHase* also decreased the developmental time of larvae and larval weight. Thus, research has demonstrated that SAHase is essential in JH biosynthesis in insects [72]. Oral ingestion of dsRNA to target *LdJHDK* depicted a significant downregulation of the target gene, and an increase in JH titer and *LdKr-h1* mRNA level. Adult emergence was significantly affected by silencing of this gene. This research suggested that this gene is connected with JH degradation and thus can be used in accordance with JH mimics. Concluding, these dsRNAs targeting *LdSAHase* can be used as synergists with some JH mimics [73].

Vacuolar-type ATPases (vATPases) are ATP-driven proton pumps with a variety of physiological functions in insects, which are important for insect survival [74]. *Its* levels increase significantly during immature stages to the final instar and then start decreasing in pupae and are upregulated again during the adult stage. Higher expression was observed in the digestive tract as compared to the rest of the organs [75,76]. Feeding with a bio-assay using dsRNAs targeting *LdATPaseE1* and *LdATPaseE2* decreased their expression in larvae by 85% and 55%, respectively. Larval development and survival rate were significantly reduced. Additionally, contact bioassays with cypermethrin, endosulfan, fipronil, and butane-fipronil have been demonstrated to increase the expression of *LdATPaseE*. It depicts that targeting vATPase subunit E can be a promising target in the management of the CPB [77]. Furthermore, dsRNA targeting the vATPase subunit *E* can be helpful as a synergist with various insecticides [78,79].

The 20-hydroxyecdysone (20E) hormone is a key player involved in ecdysis and metamorphosis in insects. Two 20E-related genes, i.e., *LdFTZ-F1-1* and *LdFTZ-F1-2,* were targeted via a feeding assay in the CPB. This assay resulted in a decrease in the level of ecdysteroidogenesis genes, a decreased 20E titer, and downregulated the 20E receptor gene expression, hence, resulting in the failure of pupal formation [80]. Three clones of CPB ecdysone-induced protein *75* (*LdE75*) primarily consisting of *LdE75*A, B, and C, highly expressed at the termination and initiation of each molt, were targeted via a feeding bioassay. The ingestion of ds*E75-*1 and ds*E75-*2, containing a conserved sequence of the three analogues significantly silenced these *LdE75*s and ceased their development. Knocking down *LdE75*s also affected the expression of genes involved in JH biosynthesis, and enhanced JH tit and the expression of genes associated with JH. This research demonstrated that *LdE75*s have an important role in metamorphosis and thus can be used as synergists with IGRs [81].

Ryanodine receptors (RyRs) regulate various physiological processes such as neurotransmitter release, muscle contraction, and hormone secretion [82]. A higher level of *LdRyR* expression can be observed at larval stages, especially in the 4th instar, and in the adults of many insect pests [83,84,85]. A feeding bioassay using double-stranded RNA targeting *LdRyR* successfully downregulated the target gene in the CPB adults and larvae. Research has shown that *LdRyR* is important in the functioning of the ryanodine receptor in CPB. The mortality due to downregulation of *LdRyR* can increase to 48.2%. They can be important targets for increasing the life of active insecticides such as chlorantraniliprole against the CPB [86].

Cuticular pigmentation and hardening are controlled by enzymes encoded by the *laccase2* gene [87]. Both injection method and feeding bioassay were used for inserting the dsRNA targeting *laccase2* gene in the CPB. Significant phenotypic changes were observed due to microinjections containing dsRNA as compared to the one introduced via a feeding bio-assay. There was no significant change in RNAi genes due to the introduction of dsRNA, despite the fact that various genes associated with the RNAi pathway were over-expressed [88]. Standardizing the delivery methods for RNAi can be a promising method to study insect host interactions. Moreover, this gene can be useful if used as a synergist with chitin synthesis inhibitors such as diflubenzuron, hexaflumuron, and teflubenzuron [89].

The mevalonate pathway can be an important target for gene silencing as it has a crucial role in the biosynthesis of various crucial proteins important for insect growth, reproduction, communication, and immunity [90]. Ten important genes encoding acetoacetyl-CoA thiolase (*LdAACT1*and *LdAACT2*), mevalonate kinase (*LdMevK*), phospho-mevalonate kinase (*LdPMK*), hydroxymethylglutaryl (HMA)-CoA synthase (*LdHMGS*), farnesyl pyrophosphate synthetase (*LdFPPS*), mevalonate diphosphate decarboxylase (*LdMDD*), HMG-CoA reductase (*LdHMGR1* and *LdHMGR2*), and isopentenyl-diphosphate isomerase (*LdIDI*) were identified in the CPB. Nine of these genes (except for *LdAACT1*) were found in larvae and adults. The knock-down of *LdJHAMT* significantly downregulated the expression level of these nine genes. The expression of these nine genes was also decreased due to the ingestion of JH for the activation of JH signaling. Concluding, targeting these genes can be helpful for resistance management of JH mimics [91].

Nicotinic acetylcholine receptors (*nAChRs*), which belong to the Cys-loop ligand-gated ion channel superfamily, including 5-hydroxytryptamine 3(5-HT3), glycine, and gamma-aminobutyric acid (GABA) receptors, mediate excitatory cholinergic neurotransmission in the central nervous system of insects. Native *nAChR*s of insects are homopentamers of α subunits, or heteropentamers of α and β subunits. In total, 10–16 types of *nAChR* subunit genes have been identified in different insects [92,93]. Four new nAChR subunits *Ldα3*, *Ldα6*, *Ldα10*, and *Ldβ1* obtained from the CPB were targeted. They are highly expressed, during all growth stages, in the head, thorax, and abdomen. Feeding with double-stranded RNA targeting *Ldα1* (ds*Ldα1*) significantly decreased the expression of *Ldα1* in CPB adults and larvae. A bioassay conducted on ds*Ldα1*-treated adults significantly decreased the susceptibility to neonicotinoids in adults. Concluding, *Ldα1* encoding nAChR has an important role in the detoxification of imidacloprid and thiamethoxam against CPB. Therefore, it can be used to break the resistance or tolerance of CPB to neonicotinoids [94].

Several imidacloprid-resistance-conferring genes are documented. Among these genes, the cuticular protein (*CP*) plays a vital role in penetration resistance [95], cytochrome P450 monoxygeneases (*P450*) work during phase I reactions [96], gluthione synthetase (*GSS*) is the key player in phase II reactions [97], and ATP binding cassettes (ABC transporters) are involved in the ATP-dependent transport of various substances, including toxins [98]. Where, UDP-glycosyltransferases (UGTs) play a role in the catalysis of a sugar donated with lipophilic molecules to produce water-soluble compounds which can be excreted easily later [99]. A set of these three over-expressed imidacloprid-resistance-conferring genes, i.e., CP, GSS, and P450, were selected for RNA interference experiments by the injection method. Significant knock-down of genes encoding enzymes in a resistant CPB population was carried out. The resistance to imidacloprid was significantly decreased in treated populations, which suggests the utilization of these dsRNA as synergists with imidacloprid and other neonicotinoids [100]. The expression of other imidacloprid resistant genes such as cytochrome P450s (*CYP6BQ15*, *CYP4Q3*, and *CYP4Q7*), one ATP binding cassette (ABC) transporter (*ABC-G*), one esterase (*EST1*), and two UDP-glycosyltransferases (*UGT1* and *UGT2*) was decreased by conducting a feeding bioassay with dsRNA. Additionally, the knock-down of imidacloprid-resistance-conferring genes (*CYP4Q3* and *UGT2*) significantly increased the efficacy of imidacloprid to resistant beetles, indicating that these targets can be successfully used for utilizing RNAi as a synergist with imidacloprid [101]. Similarly, four cytochromes p-450 genes, i.e., *CYP6BJ*, *CYP6BJ1v1*, *CYP9Z25*, and *CYP9Z29* were successfully silenced. These targets can be utilized to prolong the efficacy of neonicotinoids and plant defense against the CPB [102].

The nucleases in some polyphagous insects such as the CPB can be a possible reason for degradation and thus failure of dsRNA in many insects [103]. Two important nucleases were isolated from the gut of the CPB followed by identification and categorization. The silencing of nuclease genes in adults decreased the resistance of this insect towards dsRNA, resulting in enhanced protection of plants. In conclusion, silencing of nuclease activity can cause a synergistic effect for the activity of other dsRNA and many stomach poisons [104], as shown in Table 2.

### Future Possible Targets

Though several resistance-associated genes have been documented, most of them have not yet been explored for their potential use in RNAi silencing. These targets for the CPB can be further considered in future studies. Proteinase inhibitors (PIs), key players in anti-nutritional activities in the digestive tract of insects, are a main part of plant natural defense [105]. Larvae detoxify PIs being synthesized in potato leaves via substitution of inhibitor-sensitive digestive cysteine proteases with inhibitor-insensitive cysteine proteases. Enzymes involved in digestion are very important in this regard. These are the initial barriers to all kinds of plant secondary metabolites and insecticides. These are also involved in the detoxification of food-related toxins. Important digestive enzymes include cysteine proteases, intestains D, intestains E, cellulases, serine proteases, and an endopolygalacturonase [106].

Insect cytochrome p-450 (CYPs) genes have a significant role in making insects capable of survival in a chemically diverse environment. Some of these CYPs detoxify a large variety of plant metabolites, synthetic insecticides, and/or other environmental chemicals [107,108]. While some of them have a very crucial role in physiological functions of insects such as the synthesis of JH and molting hormone [109]. CYPs can also degrade pheromones in some insects, which are a source of alteration in insect behavior and chemical communication [110]. Furthermore, catalyzation and hydroxylation of fatty acids for the synthesis of physiologically important biochemicals is carried out by CYPs [111].

Based on the CPB transcriptome dataset and the GenBank sequences, 70 novel carboxylesterases and 2 acetylcholinesterases were found. The 72 members belong to a multifunctional carboxylesterase/cholinesterase superfamily (CCE). All CCEs can be categorized into three main phylogenetic categories, which include dietary/detoxification, neurodevelopmental classes, and hormone/semiochemical processing. The numbers of CCEs in the CPB reported till now are: 52 (dietary/detoxification enzymes), 12 (hormone/semiochemical processing enzymes), and 8 (neurodevelopmental enzymes). The dietary/detoxification class can be further divided into two categories: α-esterase type and coleopteran xenobiotic metabolizing CCEs. The hormone/semiochemical processing enzymes include: β- and pheromone CCEs, exosekeleton-related CCEs, and juvenile hormones. Acetylcholinesterase, neurotactin, neuroligin, gliotactin, glutactin, and many others are the main neurodevelopmental CCEs. Among the 70 novel CCE genes, *KM220527, KM220538*, KM220541, *KM220542, KM220554, KM220561, KM220564, KM220566,* and *KM220578* were cyhalothrin-inducible while *KM220527*, *KM220530, KM220541*, *KM220566,* and *KM220576* were fipronil-inducible [112,113]. Nine *Cyp* genes, i.e., *Cyp12H2*, *Cyp6BH2*, *Cyp6BJ1*, *Cyp6BQ17*, *Cyp6EG1*, *Cyp6EH1*, *Cyp6EJ1*, *Cyp4BN13v1,* and *Cyp4BN15*, were highly expressed in a pyrethroid-resistant population [111]. *LdGSTe2a*, *LdGSTe2b*, *LdGSTo5,* and *LdGSTt1* were significantly overexpressed after exposure to each of the three insecticides, cyhalothrin, fipronil, or endosulfan [114].

Other closely related genes such as *Ldα3*, *Ldα9,* and *Ldβ1* were significantly over-expressed in larvae exposed to insecticides. Where, *Ldα4*, *Ldα7,* and *Ldα9* are the nAChR subunit genes that play a role in producing resistance to neonicotinoids [115]. Peaks in imidacloprid resistance have been reported due to various mechanisms of resistance such as mixed-function oxidases [17], cytochrome p450, and cuticular protein transcripts [116].

Basic helix–loop–helix (bHLH) transcription factors have important roles in functions such as cell proliferation, determination, differentiation, maintenance of the cell cycle, and response to different kinds of stresses. The categorization and characterization of bHLH members is the first step. Through transcriptome analysis, 49 bHLH members have been identified. All *Ld*bHLH members were defined by their names and families according to various phylogenetic analyses with bHLH homologues of *Drosophila melanogaster*, *Apis mellifera*, *Bombyx mori*, and *Tribolium castaneum*. These results have provided the base for using them in combination with a few JH mimics targeting bHLH members [117] (Table 2).

**Table 2:** Target genes of dsRNA which can be used to synergize insecticide(s).

Gene Family	Target Gene of DsRNA	Compatible Insecticide(s)	References
Juvenile hormone pathway	*LdSAHase*	Juvenile hormone mimics	[68]
Juvenile hormone related gene	JHDK	Juvenile hormone mimics	[73]
Vacuolar ATPases	*LdATPaseE1* and *LdATPaseE2*	Multiple groups of insecticides	[77]
20-hydroxyecdysone genes	*LdFTZ-F1-1* and *LdFTZ-F1-2*	Juvenile hormone mimics	[81]
Ecdysone-related genes	*LdE75*A, B, and C	Ecdysteroid agonists	[81]
Ryanodine receptor	*LdRyR*	Chlorantraniliprole	[86]
Sclerotization gene	Laccase2	Chitin synthesis inhibitor	[88]
Mevalonate-pathway-related gene	*LdJHAMT*	Juvenile hormone mimics	[91]
*nAChR* genes	*Ldα3*, *Ldα6*, *Ldα10*, and *Ldβ1*	Neonicotinoids	[94]
Cuticular protein	CPH30	Neonicotinoids	[100]
	*CYP6BQ15*, *CYP4Q3*, and *CYP4Q7*	Neonicotinoids	[101]
Cytochrome P450s	*CYP6BJ*, *CYP6BJ1v1*, *CYP9Z25*, and *CYP9Z29*	Neonicotinoids and plant secondary metabolites	[102]
Random	Nucleases	Stomach poisons and other dsRNA	[104]
Digestive genes	Cysteine proteases, intestains D, intestains E, cellulases, and serine proteases	Plant proteins/protease inhibitors	[106]
Carboxylesterase/cholinesterase superfamily	*CCE* genes	Pyrethroids, phenylpyrazole	[112]
Glutathione synthetase	LdGSTs	Neonicotinoids, pyrethroids, organophosphate, and phenylpyrazole	[114]
Basic helix–loop–helix genes	*Ld*bHLH	Hydroprene, methoprene, and pyriproxyfen	[117]

Various researchers have shown that dsRNAs can resist high temperatures (around 80 °C) and their spray can resist field conditions such as high temperatures and UV lights [118,119], where the messenger RNA (mRNA) level, exploiting a sequence-dependent mode of action, has made it unique in potency and selectivity compared to conventional agrochemicals [120].

## 5. Conclusions

Over-reliance on insecticides also causes human-health hazards and environmental pollution. Every chemical insecticide, either currently used for the CPB or in the future, will ultimately result in resistance. Such resistance involves a variety of genes and metabolic enzyme systems associated with them. To reduce the amount of insecticide application and extend the life of available insecticides, older chemicals can be used with eco-friendly plant-based or RNAi-based synergists against resistant insect pests. Though, some possible challenges can be faced regarding the extraction of plant products for their use as synergists. Additionally, proper selection and the medium of introducing RNAi in the field can require further research on these aspects, and possible implementation in the field can significantly decrease our cost of developing new insecticides.

## Figures and Tables

**Table 1:** Insecticide(s) synergized by addition of different plant-based synergists against different insect pests.

Category of Plant-Based Synergist	Name of Plant-Based Synergist(s)	Synergized Insecticide(s)	References
Plant oils	D-carvone, Myristicin, Apiol	Carbamates (carbaryl, carbofuran, parathion, etc.)	[37,38,39,40,41,42,43,44]
	Karanjin and Pongamol	Pyrolan, carbaryl, endrin, or heptachlor	
	Sesame oil	Deltamethrin, cypermethrin, and fenvalerate	
	Neem oil, Citronella oil	Deltamethrin, cypermethrin, and fenvalerate	
	Pongamia oil	Pyrolan, carbaryl, endrin, heptachlor, and cypermethrin	
	Dillapiol	Carbamates (carbaryl, carbofuran, parathion etc.), neem, rotenone, toosendanin, and *Annonasq uamosal*	
	Cottonseed oil, linseed oil, safflower oil, pundi oil, honge oil and sesame oil	Fenvalerate, deltamethrin, and cypermethrin	
	Karanj oil	Deltamethrin, cypermethrin, fenvalerate, and eucalyptus oil	
Alkaloids	Three isomers of austrospicatine-type taxoids	Pyrethroids	[45,46,47,48,49]
	Six semi-pure fractions of flindersiamine	Pyrethrins	
	Taxifolin and quercetin	Guthion	
Phenolics	Phenolic compounds extracted from maple leaves	Rutin, kaempferol, juglone, or quercitrin, gramine, quinine, glaucine, and quillaja saponins	[50,51,52,53,54]
Terpenoids	Thymol, citronellal, and R-terpineol	*trans*-Anethole	[55,56,57,58,59,60,61,62,63]
Miscellaneous	Phyto-extracts of Surattense nightshade plant	Cypermethrin	[26,64]
	Leaf extracts of *Jatropha gossypifolia* and *Melia azedarach*	Cypermethrin	

## Data Availability

The study did not report any data.
